# Novel Algorithms Reveal Streptococcal Transcriptomes and Clues about Undefined Genes

**DOI:** 10.1371/journal.pcbi.0030132

**Published:** 2007-07-06

**Authors:** Patricia A Ryan, Brian W Kirk, Chad W Euler, Raymond Schuch, Vincent A Fischetti

**Affiliations:** Department of Bacterial Pathogenesis and Immunology, Rockefeller University, New York, New York, United States of America; Washington University in St. Louis, United States of America

## Abstract

Bacteria–host interactions are dynamic processes, and understanding transcriptional responses that directly or indirectly regulate the expression of genes involved in initial infection stages would illuminate the molecular events that result in host colonization. We used oligonucleotide microarrays to monitor (in vitro) differential gene expression in group A streptococci during pharyngeal cell adherence, the first overt infection stage. We present neighbor clustering, a new computational method for further analyzing bacterial microarray data that combines two informative characteristics of bacterial genes that share common function or regulation: (1) similar gene expression profiles (i.e., co-expression); and (2) physical proximity of genes on the chromosome. This method identifies statistically significant clusters of co-expressed gene neighbors that potentially share common function or regulation by coupling statistically analyzed gene expression profiles with the chromosomal position of genes. We applied this method to our own data and to those of others, and we show that it identified a greater number of differentially expressed genes, facilitating the reconstruction of more multimeric proteins and complete metabolic pathways than would have been possible without its application. We assessed the biological significance of two identified genes by assaying deletion mutants for adherence in vitro and show that neighbor clustering indeed provides biologically relevant data. Neighbor clustering provides a more comprehensive view of the molecular responses of streptococci during pharyngeal cell adherence.

## Author Summary

Microarray technology is commonly used to reveal genome-wide transcriptional changes in bacterial pathogens during interactions with the host. Clustering algorithms, which group genes with similar expression patterns, facilitate microarray data organization and are based on assumptions that co-expressed genes share common function or regulation; however, clustering solely by co-expression may not reveal all of the information contained in bacterial array data. We introduce neighbor clustering, a new tool for analyzing bacterial gene expression profiles, which distinguishes itself from other programs by incorporating details unique to the architecture of bacterial chromosomes into the analysis. Neighbor clustering combines two informative characteristics of bacterial genes that share common function or regulation—(1) similar expression profiles and (2) physical proximity on the chromosome—and extracts statistically significant clusters of gene neighbors that are potentially related by function or regulation. We present the analysis of microarray data from group A streptococci during adherence to human pharyngeal cells, the first overt infection step. We show that neighbor clustering identifies more differentially expressed genes than rigorous statistical analyses alone, and can provide functional clues about unknown genes. We extended the analysis to include a previously published streptococcal array study to demonstrate the applicability of the method.

## Introduction

Microarray technology is now commonly used to reveal genome-wide transcriptional changes in bacterial pathogens during interactions with the host. Several factors, however, limit the power of such analyses, including inadequate statistical analysis and insufficient sample replication, both of which do not account for experimental variability, and often result in arbitrary thresholds for significance [1,2]. In addition, unknown bacterial genes can confound the interpretation of expression profiles, restricting many microarray studies to the differential expression of well-characterized genes.

Several methods are available to organize gene expression profiles and to assist in extracting functional or regulatory gene information from microarray datasets. Clustering algorithms group genes by similarities in expression patterns, based on the assumption that co-expressed genes share common function or regulation [3,4]; however, clustering solely by co-expression patterns may not reveal a considerable amount of information contained in array data. These methods often: (1) produce unreliable data by missing known gene members of biological pathways; (2) fail to distinguish truly related gene clusters from coincidental groupings; and (3) identify clusters containing only unknown genes that may lack either common function or regulation, a considerable limitation for genomes containing a large percentage of undefined genes [1,2]. Because no tools exist to interpret unknown gene clusters or to assess their significance and completeness, a significant portion of bacterial expression profiles are not interpretable using current clustering methods.

We introduce neighbor clustering as a new tool for analyzing bacterial microarray data that addresses some of these limitations by incorporating the physical position of genes on the bacterial chromosome into the analysis of expression data. Information about gene function and regulation is stored intrinsically in the bacterial genome structure, as genes with common function or regulation tend to be physically proximate on the chromosome and often linked as operons [5,6]. We incorporated these positional data into a series of neighbor clustering algorithms, named GenomeCrawler, that identifies groupings of potentially related genes from array data by combining two informative characteristics of bacterial genes that share common function or regulation [3–6]: (1) similar gene expression profiles (i.e., co-expression); and (2) physical proximity of genes on the chromosome. The algorithms also recalculate the statistical significance of each gene as a member of a particular cluster, as well as the significance of each resulting grouping as a whole, to ensure accuracy of cluster assignments. This process ultimately identifies significant clusters of co-expressed gene neighbors that likely share common function or regulation.

We used this approach to analyze microarray expression data from group A streptococci *(Streptococcus pyogenes)* during adherence to human pharyngeal cells, the first overt infection step [7]. The ability of all bacterial pathogens to infect the human host depends upon coordinated regulation of diverse gene sets that are required for survival in host environments. Although recent microarray studies have highlighted the molecular responses of streptococci in relevant host conditions [8–10], characterizing differentially expressed loci during pharyngeal cell adherence is critical for understanding the molecular basis for host colonization. Studies from our laboratory [11,12] and others [13] have demonstrated that in vitro association with pharyngeal cells results in streptococcal phage induction and the increased expression of phage-encoded virulence factors. Although the mechanisms mediating these responses are not known, the results of these studies indicate that streptococci sense and, on a transcriptional level, respond to various signals and cues in the pharyngeal cell environment.

We undertook the present study to understand and to assess more accurately the genome-wide transcriptional responses of streptococci during one of the earliest recognized stages of infection, namely adherence to human pharyngeal cells. We compared data generated before and after neighbor clustering to show that this method provides a more comprehensive view of transcription by: (1) identifying more differentially expressed genes than even traditional, rigorous statistical analyses; (2) reconstructing intact biological pathways that statistical significance analysis could not reconstruct; and (3) providing preliminary insight and clues about the function or regulation of uncharacterized genes by associating their co-expression with physically proximate, functionally defined genes.

## Results/Discussion

### Adherence-Mediated Differential Expression

We developed spotted oligonucleotide arrays of the S. pyogenes SF370 (an M1 serotype) genome [14] and compared the transcriptomes of streptococci that adhere to Detroit 562 human pharyngeal cells to non-adherent (“associated”) streptococci within the same experiment. Adherence assays were performed as described [15] with modifications to minimize eukaryotic cell disruption. We replicated experiments independently and used dye-swaps to incorporate biological and technical variation [16,17]. Following filtering and normalization [18,19], we analyzed data from four biological replicates [16] with robust summary statistics [20], Bayesian statistics [21,22], and permutation algorithms [19] to identify genes differentially expressed with significance during pharyngeal cell adherence.

This analysis identified 79 genes (4% of the genome) exhibiting statistically significant fold changes in expression (*P*_F_ value < 0.05) during adherence from 1,769 open reading frames represented on the array (Table 1). We refer to such genes as “differentially expressed.” We present the entire dataset from all experiments as Table S1. Genes demonstrating upregulation (*n* = 45) and downregulation (*n* = 34) included virulence factors, prophage-encoded transcripts, metabolic genes, and transcriptional regulators (Table 2). Undefined or hypothetical genes comprised 27% of differentially expressed genes (*n* = 21; 11 chromosomally encoded genes, ten phage-encoded genes).

**Table 1:**
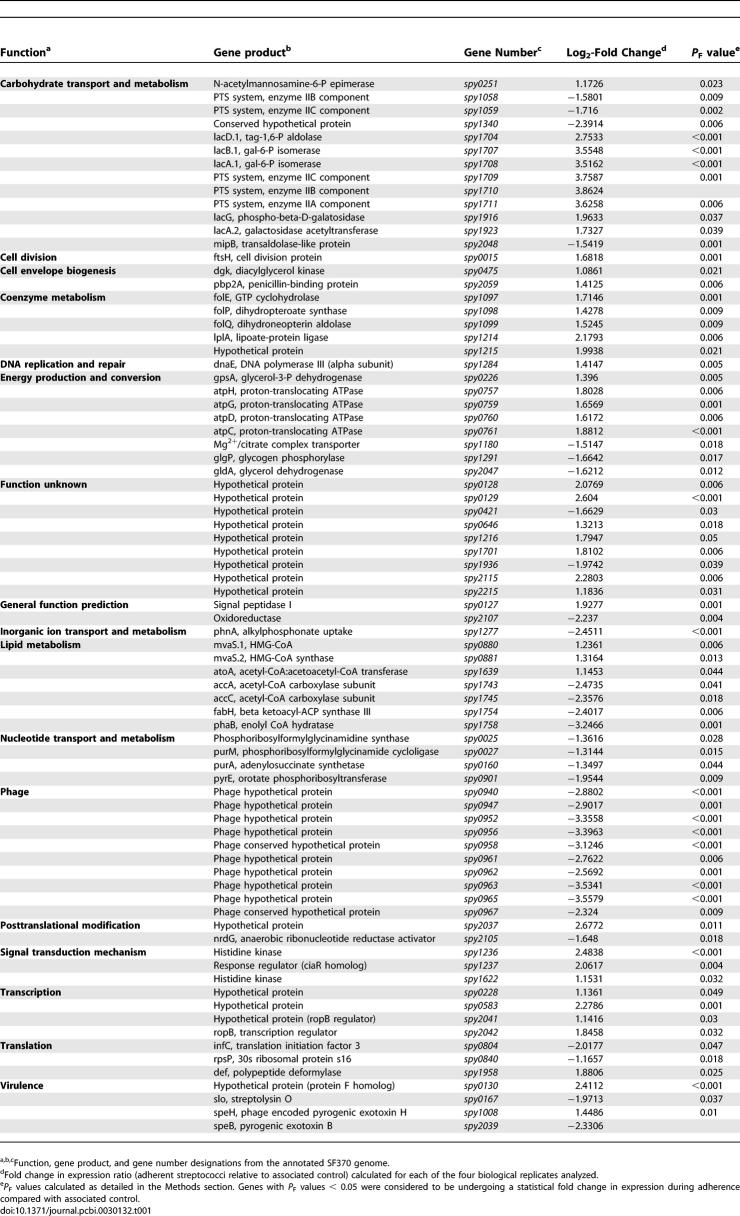
Summary of Streptococcal Genes Exhibiting Significant Changes in Expression during Adherence to Pharyngeal Cells Compared with Associated Streptococcal Control

**Table 2:**
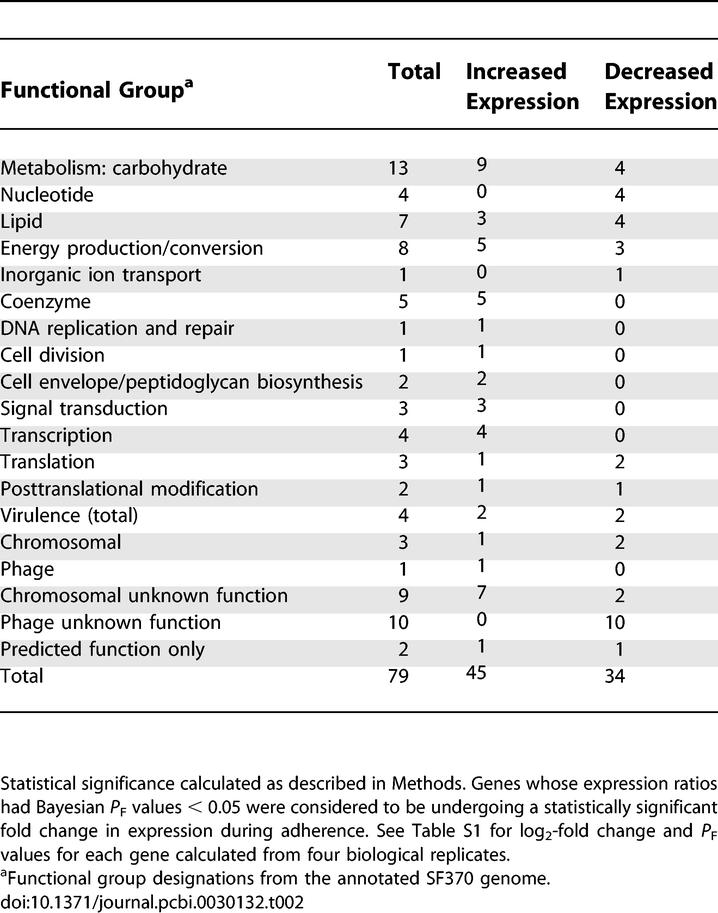
Functional Categories of Streptococcal Genes Exhibiting Significant Changes in Expression during Adherence to Pharyngeal Cells Compared with Associated Streptococcal Control

### Verification by Quantitative Real-Time PCR

We conducted TaqMan (qRT-PCR) analysis [23] of 11 differentially expressed genes to validate selected microarray hybridization results (see Table S2 for genes and primer–probe sequences). Five genes chosen for validation demonstrated statistically significant fold changes in expression by microarray analysis (*P*_F_ value < 0.05; two upregulated, three downregulated). The remaining six genes (four upregulated, two downregulated) did not have significant *P*_F_ values, but were statistically significant as members of particular neighbor clusters in subsequent analyses (*P*_E_ < 0.05) as detailed in later sections). We averaged the data to generate a value for each gene, creating a set of 11 paired values from quantitative real-time (qRT)-PCR and microarray analyses (Table S3). Results of standard linear regression analysis demonstrated a strong positive correlation (*r* = 0.9) between data obtained using the different techniques (see Figure S1).

### Virulence Factors

Streptococci elaborate several factors implicated in infection, including surface-exposed adhesins and secreted toxigenic proteins (reviewed in [7,14,24]). The initial statistical analysis identified four differentially expressed virulence genes (Tables 1 and 2). Genes encoding streptolysin O *(slo* or *spy0167)* and the SpeB protease *(spy2039)* were downregulated, while genes encoding pyrogenic exotoxin H *(speH* or *spy1008)* and a putative fibronectin-binding protein *(spy0130)* were upregulated. We verified the differential expression of *spy2039* and *spy0130* by qRT-PCR.

The downregulation of virulence loci during presumably inappropriate stages of infection was not surprising. Streptolysin O is a cytotoxin that damages human tissue and increases host cell cytotoxicity [7,25]. The resulting cellular damage, particularly to polymorphonuclear leukocytes [26], decreases internalization and subsequent intracellular killing of streptococci [27]. Based on its downregulation during adherence, we infer that *slo* was transcribed during pre-adherence associations, perhaps, as previously reported, to protect streptococci from phagocytic killing in vivo [27]. However, once adhered, our data suggest that streptococci downregulate production of this cytotoxin, presumably to prevent further host tissue destruction that could interfere with adherence.

SpeB (encoded by *spy2039*) is a multifunctional cysteine protease implicated in numerous infection strategies [28,29]. Although few studies have examined gene expression patterns during adherence, SpeB production (as detected by Western blot analysis) decreases during co-culture with human peripheral blood mononuclear cells [30] and in a mouse infection model [31]. When SpeB expression is limited, several streptococcal proteins necessary for adherence remain intact [24,32,33]; thus, decreased SpeB production (as indicated here) may promote pharyngeal cell attachment. Furthermore, SpeB abolishes internalization (following adherence) of certain streptococcal strains by epithelial cells (including Detroit 562 cells), a process mediated in part by the fibronectin-binding protein F [34,35]. We observed significant upregulation of the gene *spy0130,* encoding a protein recently found to be associated with the production of surface-exposed pili on strain SF370 [36]. The protein shares 60% sequence similarity to protein F, suggesting that it may coordinate a similar internalization mechanism or may be involved directly in adherence (discussed later in detail). *SpeB* downregulation also coincides with increased expression of pyrogenic exotoxins [33,37] that reportedly increase streptococcal survival in vivo. We observed that the exotoxin-encoding *speH* gene [38] was upregulated. Taken together, our results agree with previous reports on SpeB production during host cell interactions, suggesting that decreased expression may promote streptococcal adherence (by preventing proteolytic degradation of key virulence factors or adhesins), enhance internalization (perhaps through a fibronectin-mediated pathway), and increase survival (through increased pyrogenic exotoxin production, discussed below).

### Phage-Encoded Genes

SF370 contains one inducible prophage (370.1) and three defective prophages (370.2, 370.3, and 370.4) that produce no infectious phage [39]. We identified 11 differentially expressed phage 370.2 genes, suggesting that this defective phage is not transcriptionally silent (Table 1). The *speH* gene *(spy1008)* was induced, and the remaining genes, hypothetically involved in replication and regulation [39], were downregulated. The *speH* gene encodes a mitogenic exotoxin [38] reportedly induced during polymorphonuclear leukocyte phagocytosis [8] but not implicated previously in adherence.

### Allelic Replacement of *speH*

**Figure 1:**
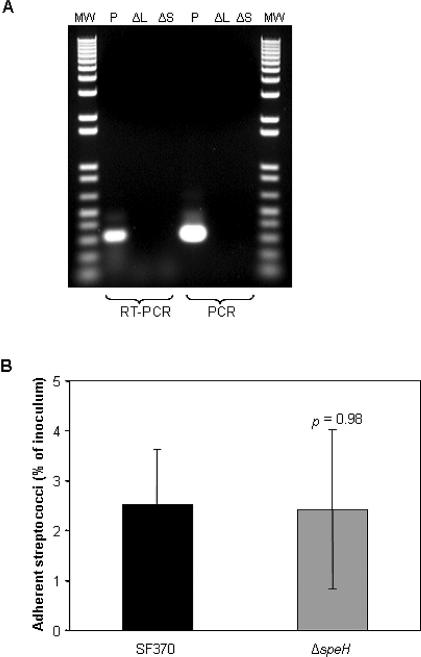
Confirmation of *speH* Deletion Mutant and Pharyngeal Cell Adherence Assay (A) Results of RT-PCR and PCR analyses of total RNA preparations isolated from mid-log (OD = 0.4) and stationary phase (OD = 1) cultures of the *ΔspeH* deletion mutant (ΔL and ΔS, respectively), and stationary phase cultures of the SF370 parental strain (P). RNA was reverse-transcribed as described in Methods. To assess genomic DNA contamination, control reactions containing Taq DNA polymerase instead of reverse transcriptase were included. cDNA products were separated on a 1% agarose gel and visualized by ethidium bromide staining. Lanes containing products from either the RT-PCR or PCR analysis are designated at the bottom of the panel. Lanes labeled MW contain 1 kb Plus DNA ladder (1 μg; Invitrogen). (B) Results of the pharyngeal cell adherence assay (detailed in Methods), comparing parental strain SF370 with the deletion mutant SF370Δ*speH* (abbreviated Δ*speH*). Adherent streptococci are reported as the percentage of total number of streptococci added as inoculum to pharyngeal cell monolayers. Statistical significance (reported as *p* value) was determined by Student's *t-*test.

Increased expression of *speH* during pharyngeal cell adherence suggests that the SpeH exotoxin is either necessary for adherence, or is a component of a downstream infection process. Adherence-mediated upregulation of *speH* is likely not the result of phage induction, as the remaining phage 370.2 genes identified in our analysis were downregulated. To determine if SpeH plays a direct role in the adherence process, we created a deletion mutant in strain SF370 (SF370Δ*speH*), which was confirmed by PCR (unpublished data) and RT-PCR (Figure 1A) and tested in vitro for adherence to human pharyngeal cells. We observed no significant difference in adherence between the wild-type (SF370) and mutant strains (Figure 1B), indicating that SpeH is not involved directly in attachment to the pharyngeal cell. The significant upregulation of the *speH* gene during adherence suggests that the gene product may function instead during a subsequent stage of infection.

### Differential Expression of Genes from Diverse Functional Categories

We identified a number of genes encoding proteins involved in housekeeping processes (such as carbohydrate and coenzyme metabolism) that were differentially expressed, indicating a shift in metabolic processes due to host cell adherence (Tables 1 and 2). For example, genes encoding proteins involved in folate biosynthesis [40] were upregulated, suggesting that certain cofactors that may be necessary during adherence were unavailable. Also upregulated were genes encoding subunits of the F0F1 ATPase [41] (discussed in more detail later), which may indicate an acid stress response to maintain cytoplasmic pH or a need to generate ATP in response to increased energy requirements.

We also identified the adherence-mediated upregulation of four transcriptional regulators (Table 1), suggestive of an adaptive response to host cell contact that is dynamic and complex. For example, RopB (encoded by *spy2042*), a member of the Rgg family of response regulators, interacts with a number of regulatory networks throughout the streptococcal genome (e.g., *mga, csrRS, sagA, and fasBCA*), affecting the transcription of numerous proteins, virulence factors, and two-component regulatory systems [42,43]. Although the delineation of genes influenced by RopB (or any identified transcriptional regulator) is beyond the scope of this study, our initial analysis did identify the upregulation of a two-component regulatory system, encoded by *spy1236–1237*. The functions of these particular loci are not yet known, and their adherence-mediated upregulation represents new targets in the study of regulators that function during host cell contact.

### Neighbor Clustering

Our initial analysis revealed the differential expression of a wide range of functionally diverse genes and provided insight into the adaptive response of streptococci to host cell contact. However, despite a rigorous statistical approach, this analysis, like many previous microarray studies, identified the differential expression of a large number of unknown genes (*n* = 21) and a number of incomplete biological pathways (e.g., F0F1 ATPase [41] and folate biosynthesis [40]) by failing to detect the differential expression of a number of known gene pathway members (Table 1). To overcome these limitations and to extract more functional information from the array dataset (including more complete biological pathways), we developed the neighbor clustering algorithms to combine the physical position of genes on the streptococcal chromosome with gene expression data. Neighbor clustering was designed to identify expanded groupings of potentially related genes from our array data by incorporating two reliable predictors of genes that share common function or regulation, namely physical proximity and similar expression profiles [5,6].

We implemented this approach by developing an algorithm with dynamic windowing (GenomeCrawler) that sequentially stepped through the microarray data and identified clusters of adjacent genes exhibiting similar fold changes in expression. Because the genome contains many possible clusters, we restricted the algorithm's search space to identify only spatially related clusters. GenomeCrawler applied a separate permutation algorithm, using the sum of each gene's *t-*statistics to calculate adjusted *P* values (*P*_K_) for each cluster, which corresponded to the probability of assembling a cluster by chance. Significance was assigned to clusters with *P*_K_ < 0.05, and the resulting groupings are listed in Table 3. Because individual genes could be members of many different significant clusters, GenomeCrawler then applied a distinct permutation algorithm to calculate the probability (*P*_C_) that a gene was clustered coincidentally. Calculation of *P*_C_ values relies on Bayes' Theorem, in which the probability of a gene's log_2_-fold change (*P*_F_ value) is combined with the cluster probability itself (*P*_K_ value). We stress that *P*_C_ reflects the significance of a gene based on its cluster context rather than a recapitulation of *P*_F_. This ensures a strong dependency between *P*_F_ and *P*_C_, preventing a gene with a relatively low log_2_-fold change from being scored as significant simply because it is clustered with a gene with a highly significant *P*_F_ value. Finally, GenomeCrawler calculated the overall significance of differentially expressed genes (*P*_E_ values) by integrating differential expression probabilities (*P*_F_) and cluster context probabilities (*P*_C_). We developed a plotting application (GenomeSpyer) that represents the chromosome as a linear molecule to visualize GenomeCrawler output, with genes displayed on the *x*-axis and their log_2_-fold change magnitudes on the *y*-axis. Applications and all datasets are available for download at http://www.rockefeller.edu/vaf/streparray.php.

**Table 3:**
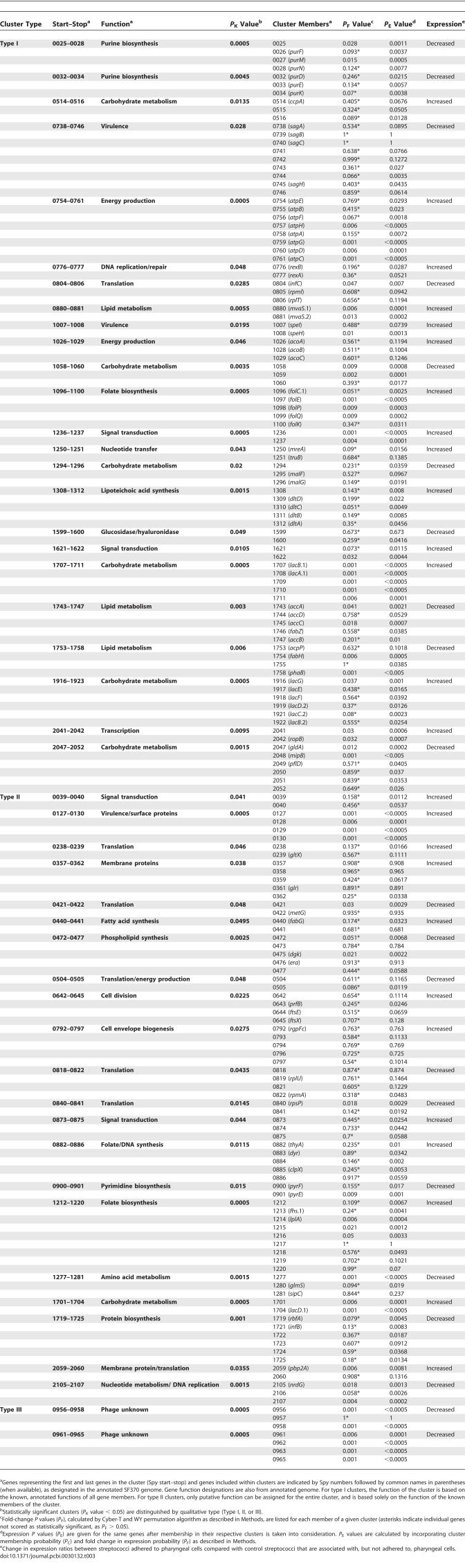
Qualifying Neighbor Clusters Identified in the S. pyogenes SF370 Genome by GenomeCrawler Analysis of Adherent Versus Associated Microarray Data

**Figure 2:**
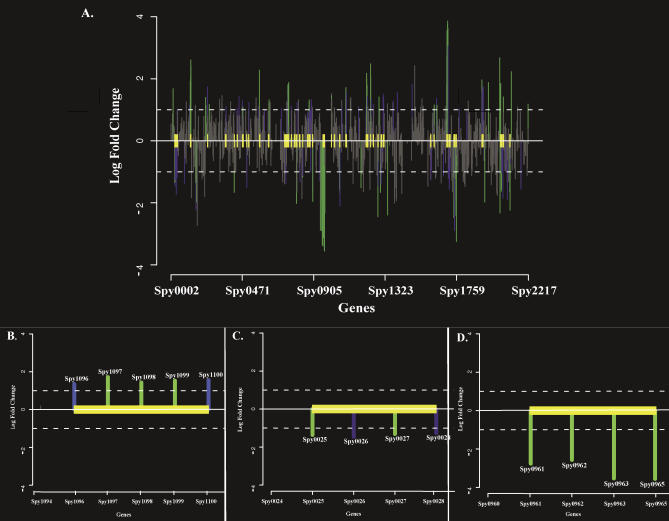
Statistically Significant Neighbor Clusters in the SF370 Genome Neighbor clusters that adhere to the definition of neighbor clusters are plotted by GenomeSpyer. Yellow boxes denote boundaries of significant neighbor clusters (*P*_K_ value < 0.05). Genes, located on the *x*-axis, are identified by their Spy numbers from the annotated SF370 genome (deleted gene numbers result during genome updates); log_2_-fold change in expression values (adherence versus associated streptococci) are indicated on the *y*-axis. Genes designated by green lines have statistically significant *P*_F_ values (log_2_-fold change *P* values < 0.05) and *P*_E_ values (expression *P* values < 0.05). Genes designated by blue lines do not have statistically significant *P*_F_ values, but as a result of membership in a designated neighbor cluster have statistically significant *P*_E_ values (*P*_E_ values < 0.05). Genes designated by gray lines do not have statistically significant *P*_F_ or *P*_E_ values. (A) Whole-genome view of 47 statistically significant neighbor clusters identified in the SF370 genome during adherence to pharyngeal cells. (B) Enlarged view of representative Type I cluster encoding folate biosynthesis genes *(spy1096–1100).* Type I clusters contain only genes of known or defined function. See text for further descriptions of all clusters. Spy numbers are indicated above the bars corresponding to each gene. (C) Enlarged view of representative Type II cluster containing *spy0127–0130.* Type II clusters contain a combination of both functionally defined and unknown gene members. (D) Enlarged view of representative Type III cluster containing phage encoded genes of unknown function *(spy0961–0965).* Type III clusters contain only genes of unknown or undefined function.

We visually inspected the resulting clusters and disqualified those that violated our neighbor cluster definition (see Methods for details). All output prior to cluster disqualifications is included for comparison (see Table S4). Of the 309 qualifying clusters (Table S5), 197 (63.8%) were composed entirely of known, functionally defined genes; however, 26 (13%) of these were incorrectly assembled, as they contained known genes that are functionally unrelated. Because we did not incorporate functional annotations of genes into the algorithms (i.e., to keep the analysis “blind”), we anticipated the possibility that some groupings could be assembled incorrectly despite the statistical framework for assigning clusters. Of the remaining 283 (91.6%) groupings, a number of differently sized clusters contained the same gene (Table S5). We report such clusters first by highest significance (lowest *P*_K_ value), then by largest number of genes. Thus, if clusters containing a particular gene were of equal significance, we report the cluster with the most gene members. This method identified 47 significant clusters containing 173 differentially expressed genes (listed in Table 3 and visualized in Figures 2 and S2–S4), a considerably larger group than could have been compiled using only the initial 79 significant genes. A total of 56 of the original 79 significant genes became components of significant clusters, whereas 23 remained unclustered.

We subdivided all clusters into three qualitative types based on the functional annotation of gene members. We present examples of Type I and II clusters: Type I clusters (*n* = 25) contained only functionally defined and functionally related genes (as reported in published studies), such as biological pathways components (Figures 2B and S2); Type II clusters (*n* = 20) included both known and unknown genes (Figures 2C and S3). Type III clusters (*n* = 2) were composed entirely of unknown genes (Figures 2D and S4), and are not discussed in detail.

### Type I Clusters: Intact Metabolic Pathways and Multimeric Proteins

We measured the performance of our algorithm by examining whether it identified gene groupings known to be functionally related (Type I clusters). Only four (16%) of 25 Type I clusters *(spy0080–0081, spy1236–1237, spy1707–1711, spy2041–2042)* could have been identified in entirety by significance analysis because all clustered genes exhibited significant differential expression (*P*_F_ value < 0.05). A total of 11 (52.4%) of the remaining 21 clusters would not have been identified in their entirety without GenomeCrawler because we initially identified significant fold-changes in only a subset of genes necessary to encode particular pathways or loci; this is intuitively unreasonable if all genes are essential for functionality. GenomeCrawler expanded these clusters to contain more genes that encode intact loci (Table 3).

For example, we initially identified (Table 1) the significant upregulation of three of the five known gene members of the folate biosynthetic pathway [40] *(spy1096*–*1100),* but GenomeCrawler identified a significant cluster containing all five genes (Table 3 and Figure 2B). We obtained a similar result for the eight-gene operon encoding the F0F1-type proton translocating ATPase [41] *(spy0754*–*0761).* The initial significance analysis identified only four *atp* genes (Table 1), but neighbor clustering identified a significant cluster containing all eight genes necessary to encode a functional ATPase (Table 3).

Each of the 11 neighbor clusters that could have been only partially identified by our initial analysis alone gained gene members after application of the algorithms and became more complete sets of functionally related genes than initially identified (Table 3). These clusters encompass various metabolic processes, including purine biosynthesis *(spy0025–0028),* lactose metabolism *(spy1916–1923),* fatty acid biosynthesis *(spy1743–1747),* lipoteichoic acid synthesis *(spy1308–1312),* and sugar phosphotransferase transport *(spy1058–1060)* [14], suggesting that specific changes occur in the streptococcal metabolic program as the bacteria adhere to human pharyngeal cells in vitro.

Notably, the remaining ten Type I clusters were composed entirely of genes that individually were not significant; however, after applying our algorithms, the combined contribution of each gene resulted in a significant cluster. For example, the nine-gene operon that spans genes *spy0738–0746* encodes streptolysin S, a potent cytolytic toxin that promotes internalization and host tissue dissemination [25,44]. Though the differential expression of the individual genes was not significant following our initial statistical analysis, GenomeCrawler identified a significant downregulated cluster containing all nine genes (Table 3). Adherence-induced downregulation of streptolysin S is consistent with its previously determined role in host cell internalization [25]; however, without neighbor clustering, expression of this operon was not evident immediately.

Although individual gene members of Type I clusters may not be statistically significant as a result of technical variability within experiments [17], the genetic structure of certain Type I operons may provide an alternative explanation. For example, the streptolysin operon encodes an internal terminator downstream of the *sagA* gene (the first gene in the operon), which modulates the abundance of particular mRNA species (e.g., *sagA* mRNA versus the polycistronic message for all nine genes) under different environmental conditions [45]. If transcription is internally disrupted by such a terminator, the abundance of the *sagA* transcript may be much greater than the polycistronic message; such disproportionate transcript levels would affect log_2_-fold change values and impact the statistical significance of individual genes within these types of clusters. Thus, in addition to helping resolve clusters that would not be easily recognized because of experimental technical variability, the neighbor clustering method may help to resolve operons with such internal terminators and regulators.

These results demonstrate that neighbor clustering effectively reconstructed a number of complete pathways and loci from processed array data. Importantly, because functional gene data are not incorporated into its algorithms, GenomeCrawler is not biased toward identifying “expected” clusters. Curating the dataset following its application may make the algorithms less user-friendly; however, the elimination of such bias is essential for this type of analysis.

### Type II Clusters

Based on the Type I cluster results, we speculated that genes contained in Type II clusters might be related by function or regulation. Type II groupings contain a combination of both known and unknown gene members and could provide preliminary clues about the function of unknown genes within a particular cluster by associating their expression with neighboring genes of known and defined function. Alternatively, co-expression of genes results from common regulation, and Type II associations may suggest shared regulatory mechanisms for clustered genes. We note, however, that despite the statistical framework with which groupings are assigned, experimental evidence is necessary to confirm functional or regulatory relatedness. We do not suggest simply assigning either based on cluster membership; rather, cluster associations may provide some preliminary functional or regulatory clues for gene members.

A total of 18 (90%) of 20 Type II clusters (Table 3 and Figure S3) may not have been identified without neighbor clustering: eight (44.4%) of 18 gained additional gene members; the remaining ten comprised genes that demonstrated significant differential expression only after applying GenomeCrawler. Only two clusters *(spy0127–0130* and *spy1701–1704)* could have been identified without neighbor clustering; however, a number of these genes were initially annotated as hypothetical proteins, so a potential relationship between the gene members may not have been readily apparent.

The upregulated *spy0127*–*0130* cluster is part of a larger genomic region known as FCT (for fibronectin- and collagen-binding proteins and T antigen–encoding loci), which spans *spy0123–0136* in the SF370 genome and encodes surface proteins and transcriptional regulators [46]. A search of both the PFAM database [47] (http://pfam.wustl.edu) and sortase database (http://www.doe-mbi.ucla.edu/Services/Sortase) predicted that *spy0129* encodes a sortase enzyme, which are transpeptidases that cleave protein substrates at conserved C-terminal motifs (often LPXTG) and then anchor these proteins to the bacterial cell wall [48,49]. Recently, it was reported that the four genes spanning *spy0127*–*0130* encode, and are responsible for, the formation of surface-localized, trypsin-resistant pili that induce protective immunity against a lethal dose of group A streptococci in a mouse model of infection [36]. This same report provided the first experimental evidence supporting the sortase prediction, indicating that the gene product of *spy0129* is responsible for the cell-wall sorting of the proteins encoded by both *spy0128* (annotated as a Cpa homolog [50]) and *spy0130* (annotated as a protein F homolog [14]). Furthermore, the *spy0128-*encoded protein is the structural backbone of the pili, and the gene product of *spy0130* may be involved in stabilizing the structure [36]. Together with the identification of this cluster by GenomeCrawler, these results prompted us to study this cluster and the contributions of the gene products to pharyngeal cell adherence.

**Figure 3:**
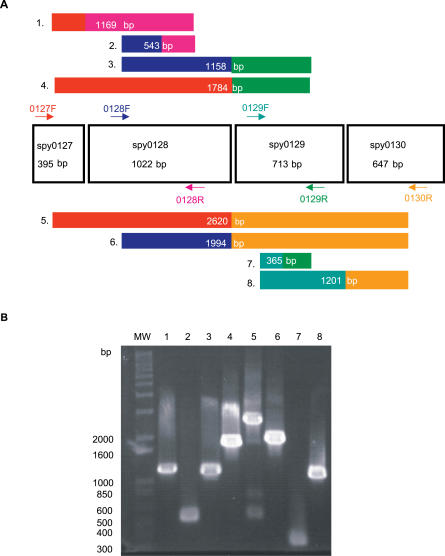
Neighbor Cluster *spy0127–0130* (A) Schematic representation of the *spy0127–0130* cluster. Gene size (bp) is indicated under the gene name, and the position of primers for the reverse transcription of streptococcal mRNA are indicated by colored arrows (forward primers, F; reverse primers, R). Eight different combinations of primers were used, and the expected sizes of the eight resulting cDNAs that would be produced if genes compose an operon are indicated in the numbered, colored boxes. cDNA box colors correspond to colors of the primer pairs that would generate each fragment. (B) A 1% agarose gel of the cDNA fragments amplified from mRNA with each primer combination. Lane numbers correspond to the numbering of the predicted cDNAs from (A). First lane of gel (MW) contains 1 kb Plus DNA ladder, and the sizes of the relevant DNA fragments in the ladder are indicated (bp).

We determined experimentally that cluster *spy0127–0130* is an operon, verifying both related function and regulation of the gene members. Reverse transcription of SF370 RNA, with primer combinations that spanned all four genes, produced cDNA fragments of sizes that could only result from a polycistronic mRNA template (Figure 3). In silico sequence inspection identified a single putative promoter sequence upstream of *spy0127* (see Table S6)*.* Although GenomeCrawler is not an operon-identifying algorithm, these results show that it could (1) identify this commonly regulated gene cluster and (2) define the cluster boundaries, excluding other proximate genes, such as an additional sortase-encoding gene, *spy0135.*

### Allelic Replacement of *spy0129*

**Figure 4:**
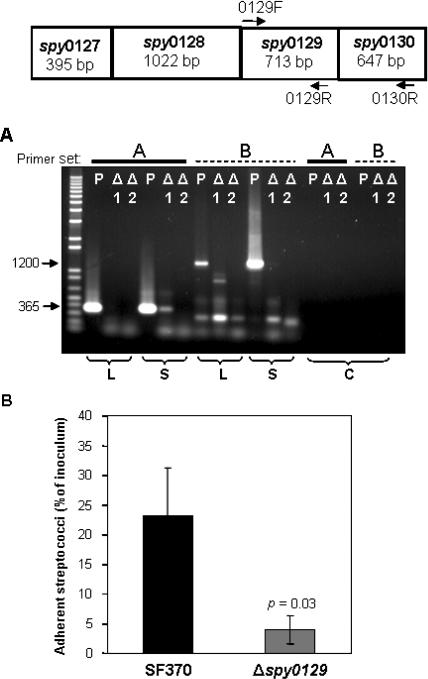
Confirmation of *spy0129* Deletion Mutant and Adherence Assay Top panel is a schematic representation of the *spy0127–0130* cluster. Position of primers for RT-PCR are indicated by arrows. (A) Results of RT-PCR analysis on mRNA from two putative deletion mutants (Δ1 and Δ2) and the parental SF370 strain (P). mRNA was isolated from mid log-phase or stationary phase cells (indicated below panel) and reverse-transcribed with two primer combinations, which are indicated at the top of the lanes as primer set A (0129F–0129R) and primer set B (0129F–0130R). cDNA products were separated on a 1% agarose gel and visualized by ethidium bromide staining. The expected sizes of resulting cDNAs from SF370 using primer set A is 365 bp and using primer set B is 1200 bp. Control reactions (C) containing mid-log phase mRNA and Taq DNA polymerase instead of reverse transcriptase are indicated. Lane 1 contains 1 kb Plus DNA ladder (1 μg; Invitrogen). (B) Results of the pharyngeal cell adherence assay (detailed in Methods), comparing parental strain SF370 to the *spy0129–0130* isogenic mutant, SF370Δ*spy0129*.2 (abbreviated as Δ*spy0129*). Adherent streptococci are reported as the percentage of total number of streptococci added as inoculum to pharyngeal cell monolayers. Statistical significance (reported as *p*-value) was determined by Student's *t-*test.

We created a *spy0129* deletion mutant in strain SF370 (SF370Δ*spy0129*) to determine if genes contained within the *spy0127–0130* cluster were directly involved in adherence to pharyngeal cells. We posited that a deletion in the *spy0129* sortase gene may have the greatest overall effect on the production and processing of the gene products of this cluster, since both the *spy0128* and *spy0130* gene products do not localize to the cell-wall surface in the absence of the sortase enzyme [36]. Allelic replacement created two putative deletion mutants; however, RT-PCR analysis (Figure 4A) revealed that only one such clone (SF370Δ*spy0129*.2) was a true knock-out for the *spy0129* gene and useful for further study. Because the gene cluster is also an operon, expression of the downstream gene *spy0130,* encoding the protein F homolog/pilus protein, was also eliminated in this mutant (Figure 4A). In vitro pharyngeal cell adherence assays revealed that the SF370Δ*spy0129*.2 mutant was approximately 66% less adherent than the parental control strain, SF370 (Figure 4B; *p* = 0.03 as determined by the Student's *t*-test). These results suggest that either the *spy0130* gene product is involved directly in adherence, or that due to the elimination of the sortase, the pili, which may function in their entirety as adhesins, were not assembled on the surface of the mutant. Because the *spy0129* gene product is not expected to be found on the streptococcal surface (i.e., it lacks a cell-wall anchoring motif), it is not likely to be involved directly in adherence. We are working to produce an in-frame deletion of *spy0128* and a *spy0130* single knock-out mutant to delineate the contribution of each individual clustered gene product to adherence.

These results show that neighbor clustering is able to identify biologically relevant gene clusters. This attribute may be particularly important for datasets in which the relationship between clustered genes is not obvious, and may facilitate the organization of larger datasets into more manageable packages.

### Additional Type II Cluster Example

Another cluster, *spy1725–1719,* contained six genes that together (though not individually) exhibited significant downregulation. The genes *spy1724, spy1722, spy1721,* and *spy1719* share transcriptional order and predicted function with homologs in the *nusA*-*infB* protein biosynthesis operon of Bacillus subtilis and Escherichia coli [51]. We examined the *spy1725* and *spy1723* gene products (annotated as hypothetical proteins [14]) for similarities with known proteins that might indicate a role for these gene products in protein biosynthesis. BlastP analysis aligned the *spy1725* gene product, which has homologs in all sequenced streptococcal genomes, with the SP14.3 protein from S. pneumoniae [52] (80% sequence similarity; 67% identity). Based on structural characterization, SP14.3 is a predicted RNA-binding protein. The *spy1723* gene product has similar domain structure to the YlxR protein of *S. pneumoniae,* an RNA-binding protein implicated in transcription termination [53]. These results indicate that both genes likely encode RNA-binding proteins, in agreement with their functionally defined cluster members. Although domain and homology searches yielded the functional predictions, their membership within a protein biosynthetic cluster provided the initial indication of common function or regulation.

### Neighbor Clustering and Operons

Although neighbor clustering is not an operon-predicting method, we wanted to identify additional putative operons among the groupings since neighbor clusters by definition share certain operon characteristics (tandemly arranged genes, separated by <300 bp, with similar expression patterns). Although operon-modeling methods exist [54,55], we inspected clusters in silico for upstream regulatory elements and identified 17 candidates*,* including clusters such as streptolysin S that have been previously confirmed as operons [56]; the *spy0127–0130* grouping, which was confirmed as an operon in this study; and others that have yet to be verified (Table S6). Experimental confirmation of each candidate is beyond the scope of this study, but Northern blot and RT-PCR analyses could provide such information.

### Analysis of Previously Published Array Data

We applied the statistical analysis and the GenomeCrawler algorithms to data from a recently published streptococcal microarray study that is relevant for comparison to our own data (same streptococcal strain, similar array platform) [57]. In this study, the transciptomes of S. pyogenes strain SF370 and an isogenic mutant deficient for the Mga regulon were compared during exponential growth in culture broth. The Mga regulator is a growth-phase mediator of a number of surface-exposed molecules and secreted proteins involved in colonization and immune evasion during infection [58]. Although the authors of that study did not provide a statistical analysis of their data, we compared the published results for the magnitude and direction of fold-changes for each gene reported in this study with those obtained from our initial significance analysis of this dataset (presented as Table S7). A total of 256 genes reported in this study were also detected by our analysis, and the magnitude and log_2_-fold change were found to be in agreement for 81% of the genes. We suspect that this discrepancy results from different normalization methods used, or from different methods that were applied to analyze the ratio of signal intensities between sample and control (i.e., we analyzed the ratios of the median rather than the ratios of the mean [57]). Although the published report did not include statistical analysis of the data, we note that the statistical analysis that we performed identified four genes with significant log_2_-fold changes in expression (*P_F_* < 0.05; Table S8).

We applied the GenomeCrawler algorithms to the statistically analyzed dataset, which identified an expanded group of genes (107 versus four) contained within 36 statistically significant clusters (*P_K_* < 0.05; Table S9). These groupings included clusters of genes that have been shown previously in streptococci to be functionally related, indicating that the algorithms were performing as expected. Two of the identified upregulated clusters *(spy2009–2010* and *spy2039–2040)* encoding the well-studied virulence factors, C5a peptidase and SpeB, respectively, showed consistently large log_2_-fold changes of the genes across replicates [57]. GenomeCrawler confirmed these results by identifying both groupings as statistically significant neighbor clusters.

GenomeCrawler also identified a number of clusters that contained genes known to share common function or regulation; however, they were not as apparent in the dataset without its application. For example, the algorithm identified a significant neighbor cluster spanning *spy0711–0712.* This grouping encodes two known virulence factors, pyrogenic exotoxin SpeC and the MF2 DNase, previously shown to be commonly regulated as an operon [11]. The algorithm also identified other neighbor clusters containing genes known to be functionally related, including *spy0098–0100* (encoding the β and β′ subunits of DNA-dependent RNA polymerase), *spy2159–2160* (encoding the 50S ribosomal subunit proteins L32 and L33), and *spy0741–0746* (six of the nine streptolysin S–encoding genes) [14].

Although the analysis of this previously published dataset did not reveal as many intact biological pathways as were identified from the pharyngeal cell adherence data, the inclusion of more replicates in the analysis to increase statistical power could resolve such loci. However, these results provided further supporting evidence that the GenomeCrawler algorithms can identify (1) a larger group of genes than a rigorous statistical analysis alone and (2) biologically relevant groupings in other microarray datasets, even if they contain fewer replicates than presented in our study.

### Concluding Remarks

Although GenomeCrawler improves bacterial array analyses, it has limitations: it cannot identify regulons comprising genes dispersed throughout the genome by virtue of its design, it does not specifically interrogate single-gene operons, and it only applies to genomes with available and accurate experimental information (expression data and gene annotations). We recognize that incorporating intergenic distance and transcription direction into the algorithms would reduce processing time. Adding available clusters of orthologous groups (COG) information into a downstream processing step could decrease errors by minimizing clustering of unrelated genes.

Nonetheless, neighbor clustering provided a more comprehensive view of the transcriptome of group A streptococci during adherence to human pharyngeal cells, a critical step in the infection program of this organism. We found that even a rigorous statistical analysis of well-replicated microarray data produced a dataset that was somewhat limited, although certainly more informative than assigning arbitrary thresholds for significance. As described in other microarray reports, we had initially identified a number of incomplete biological pathways in which we did not detect the differential expression of a number of known pathway members. Neighbor clustering was able to extend the results by identifying more differentially expressed genes and reconstructing more intact biological pathways.

Neighbor clustering, despite the statistical framework with which it assigns groupings, would be valuable to microarray data analysis only if it produced biologically relevant data. Although biological testing of every identified gene or cluster is unrealistic, we provided evidence, through the creation and testing of isogenic deletion mutants and through the identification of clusters of known, functionally related genes from a published streptococcal array study, that the algorithms produce results that are pertinent to the biology of streptococci. This may be of particular importance for data in which the relationship between clustered genes is not obvious, and may facilitate the organization of larger datasets into more meaningful packages. It is also possible that GenomeCrawler (in its current form) could be used to interrogate intergenic portions of the genome (such as those encoding small noncoding RNAs or sRNAs), if probes representing such regions were included on the microarray, and experimental conditions were designed to promote their differential expression. Finally, because of the common architecture of bacterial chromosomes, the neighbor clustering algorithms may be applicable to microarray datasets from other prokaryotes.

## Methods

### Spotted oligonucleotide microarrays.

Sense strand oligonucleotides (primarily 55-mers), representing the 1,769 open reading frames in the genome of S. pyogenes strain SF370 (M1 serotype) [14] were designed and produced by Illumina (http://www.illumina.com). Oligonucleotides were spotted using a Biorobotics Tas II 6100 arrayer (http://biorobotics.org) onto Corning UltraGAPS (gamma amino propyl silane–coated) slides (Corning Life Sciences, http://www.corning.com/lifesciences), and slides were post-processed and blocked according to the manufacturer's instructions. Each oligonucleotide was spotted four times in a well-spaced configuration to generate in-slide replicates.

### Bacterial cultures.

For adherence assays, S. pyogenes strain SF370 (kindly provided by J. Ferretti, University of Oklahoma Health Sciences Center, Oklahoma City, Oklahoma, United States) was grown to late log-phase (OD_600_ = 0.7) in Todd Hewitt broth (BD Biosciences, http://www.bdbiosciences.com) containing 0.2% yeast extract (THY; BD Biosciences). Bacterial cells were washed in 0.1 M phosphate-buffered saline (PBS; pH 7.4), resuspended in minimal essential medium (MEM; Invitrogen, http://www.invitrogen.com), and incubated for 1 h at 37 °C. Glycerol (20% vol/vol) was added, and cultures were flash frozen in liquid N_2_ and stored at −80 °C. To minimize culture-to-culture variability, these stock cultures were used for all subsequent adherence and association experiments.

### Pharyngeal cell association and adherence assay.

Assays on streptococcal adherence to human pharyngeal cell line Detroit 562 were performed as described previously [15] with the following modifications. Streptococcal stock cultures were pre-incubated at 37 °C for 1 h, and 2-ml aliquots (2 × 10^8^ CFUs) were added to confluent monolayers of Detroit 562 cells grown in wells of six-well tissue culture plates (1 × 10^7^ cells/well). Co-cultures were incubated for 2.5 h at 37 °C, and the monolayers were then washed with PBS to recover associated (nonadherent) streptococci. Pharyngeal cells were treated with 0.005% trypsin–0.004% EDTA for 15 min at 37 °C to desorb adherent streptococci (90% recovery) without disrupting the eukaryotic monolayer. Trypsin treatment does not affect gene expression in adherent streptococci compared with associated bacterial control. The monolayers were washed with PBS to recover bacteria detached by the trypsin treatment.

### RNA isolation.

Recovered streptococci were washed twice in PBS and lysed with the amidase enzyme lysin [59]. Lysin was added to the bacterial samples (2 U/10^8^ CFUs) and incubated for 15 min at room temperature, which in preliminary experiments was determined to be optimum for complete streptococcal lysis. RNA was isolated immediately after lysis with a modified phenol-chloroform protocol as described previously [60]. RNA was digested with DNase I (Invitrogen), and RNA quality was assessed with the Nucleic Acid Bioanalyzer 2100 (Agilent Technologies, http://www.agilent.com).

### Synthesis of cDNA and labeling.

DNase-treated streptococcal RNA (5 μg) was reverse-transcribed using the Atlas Glass Fluorescent Labeling kit (BD Biosciences Clontech, http://www.bdbiosciences.com/clontech). Random hexamers (Invitrogen) primed the reverse transcription reaction that incorporated a 5-(3-aminoallyl)-dUTP into the first synthesized cDNA strand. cDNAs from associated streptococci and from adherent streptococci were indirectly labeled with the N-hydroxysuccinimide activated fluorescent dyes cyanine 3 (Cy3) and cyanine 5 (Cy5), respectively, as outlined in the Atlas kit. Labeled cDNA samples were purified following Atlas kit instructions.

### Microarray hybridization and image acquisition.

Four biological replicate experiments incorporating dye swaps [17] were performed to account for both biological and technical variability. Labeled cDNA samples were hybridized to the arrays in SlideHyb hybridization buffer (Ambion, http://www.ambion.com) for 16 h at 55 °C using a GeneTAC hybridization station (Genomic Solutions, http://www.genomicsolutions.com). Slides were washed twice in 0.1 × SSC, dried, and then scanned with a Scanarray 4000 scanner (GSI Lumonics, http://www.gsilumonics.com) at 10 μm per pixel resolution. The resulting images were processed using the GenePix Pro program (version 4.0; Axon Instruments, http://www.axon.com).

### Data filtering, normalization, statistical significance analysis, and calculation of *P_F_* values for individual genes.

Following image analysis, low-level processing of microarray data included probe and array quality filtering to remove probes that were saturated, that displayed a low signal-to-noise ratio, and/or that produced signal in only one dye channel. Lowess standardization [19] was performed, and robust summary statistics were applied to the standardized log_2_-fold change data for outlier control (Huber M-estimator and unbiased MAD estimator) [20]. A Bayesian-derived regularized *t*-test was implemented with the Cyber-T program for control of variance artifacts associated with low sample size [20–22]. Calculation of the *p-*value of the log_2_-fold change for each gene (*P*_F_) uses the Westfall–Young stepdown permutation algorithm [18,19] for multiplicity adjustment in place of the Bonferroni correction typically implemented in Cyber-T. Although more computationally intensive, we chose Westfall–Young over the Bonferroni correction because: (1) Bonferroni assumes independence between tests and since genes can be regulated in conjunction with one another, we preferred to avoid the assumption of independence; (2) Westfall–Young, which is based on permutation, calculates *p-*values (from *t*-test statistics) based on the actual distribution of the data itself, and no assumption of independence is required; and (3) the power of coupling a permutation algorithm with a *t*-test is that one can take advantage of the sensitivity associated with a *t-*test, while using the distribution-free nature of a randomization test.

We used the *t*-test statistics and *P*_F_ values generated in this analysis (referred to throughout the text as initial statistical significance analysis) to rank genes [61] undergoing statistically significant changes in expression (*P*_F_ < 0.05) during adherence to pharyngeal cells compared with the associated control (Table 1). Datasets resulting from each processing step are available for download at http://www.rockefeller.edu/vaf/streparray.php.

### Real-time qRT-PCR primers, probes, and plasmid standards.

We performed real-time qRT-PCR analysis (TaqMan) on 11 different genes to verify the fold-change in gene expression estimated by microarray analysis. Five of these genes exhibited statistically significant fold-changes in expression (*P*_F_ < 0.05) during adherence (two demonstrated increased expression, and three demonstrated decreased expression), and the remaining six selected genes were scored as statistically significant only when included in a significant neighbor cluster (*P*_E_ < 0.05). The list of genes, as well as the oligonucleotide primers and fluorogenic (TaqMan) probes designed using Primer Express Software (Applied Biosystems, http://www.appliedbiosystems.com) and purchased from Sigma-Genosys (http://www.sigmaaldrich.com), are provided in Table S2. Each of the 11 genes, as well as *spy0929* (endogenous reference/control gene), was amplified in its entirety from SF370 genomic DNA by PCR and cloned into pCR-TOPO plasmids (Invitrogen). *spy0929* was chosen as control due to equivalent expression between adherent and associated SF370 cultures.

### Real-time qRT-PCR and TaqMan analysis.

We used a two-step RT-PCR procedure to reverse transcribe RNA samples from two biological replicate SF370 cultures (two adherent and two associated), which were prepared as those for microarray analysis. Using SuperScript II First Strand Synthesis System for RT-PCR (Invitrogen), DNase I-treated RNA preparations (2 μg each) were separately converted to cDNA preparations with 50 ng random hexamers (Invitrogen; 45 °C, 50 min, 20 μl reactions) according to manufacturer instructions. RNA samples were reverse-transcribed in separate reactions, and no pooling of samples occurred. Control reactions without reverse transcriptase were included to confirm that genomic DNA was not present. TaqMan analysis was performed (in duplicate) with an ABI Prism 7900 sequence detection system (Applied Biosystems) using Platinum Quantitative PCR SuperMix-UDG (Invitrogen) (according to manufacturer instructions) and primer–probe pairs listed in Table S2. No-template negative controls were included. Cycling conditions, optimized with plasmid standards, were as follows: 50 °C for 2 min and 95 °C for 2 min, followed by 45 cycles at 60 °C for 45 s.

We constructed standard curves for threshold cycle (C_T_) versus copy number for each gene with known concentrations of plasmid DNA standards (10-fold dilutions ranging from 10^8^ copies to ten copies) that were subjected to the same reaction and cycling conditions and included on each reaction plate. Results were normalized with C_T_ values for the control, *spy0929.* We averaged data from duplicate reactions to produce a single value for each gene and log_2_-transformed the fold difference in the number of cDNA molecules present in adherent streptococcal samples relative to associated streptococcal samples. This created a dataset of 11 paired values from RT-PCR and microarray analyses for each gene. We performed linear regression analysis and regressed qRT-PCR data on the microarray data.

### RT-PCR of *spy0127–0130* cluster.

The sequences of forward (F) and reverse (R) primers for each of the four genes contained within the *spy0127–0130* neighbor cluster are provided in Table S2. RT–PCR generation of amplicons was performed with the SuperScript III One-Step RT–PCR system with Platinum *Taq* DNA polymerase (Invitrogen) in reaction mixtures (50 μl) containing 0.2 μM of each gene-specific forward and reverse primers and 0.1 μg of DNase-treated, purified total RNA from late-log phase cultures (OD = 0.7) of strain SF370. All remaining components were added as per manufacturer specifications. We included control reactions, in which *Taq* DNA polymerase was substituted for the reverse transcription enzyme mixture, to confirm that genomic DNA was not present in the RNA preparations. RNA was converted to cDNA (50 °C for 30 min), which was then PCR amplified in the same tube (45 cycles of the following conditions: 94 °C for 15 s, 52 °C for 30 s, and 68 °C for 2 min). Resulting DNA fragments were separated on 1% agarose gels in TAE buffer and visualized by ethidium bromide staining.

### Allelic replacement of the *speH* and *spy0129* genes in SF370.

The strategy for allelic replacement of *speH and spy0129* genes was followed as previously described [62]. Briefly, upstream and downstream DNA regions flanking both genes were separately amplified using the primer sets listed in Table S2. PCR products were treated with the appropriate restriction enzymes (New England Biolabs, http://www.neb.com) and used according to manufacturer instructions. Fragments were gel-purified (Qiaex II Gel Extraction Kit; Qiagen, http://www.qiagen.com), and the respective upstream and downstream regions for either *speH* or *spy0129* were ligated together into the allelic replacement vector pFW15 [63], creating plasmids pFW15-*speH* and pFW15-*spy0129*. To construct deletion mutants of the *speH* and *spy0129* genes, the vectors were separately electroporated into S. pyogenes SF370 [62], and transformants were selected on proteose peptone blood agar supplemented with erythromycin (300 μg/ml). Allelic replacement was confirmed by both PCR and RT-PCR analyses of total RNA extracted (as described above) from both mid-logarithmic (OD = 0.4) and stationary phase (OD = 1) bacterial cultures using gene-specific primers. Total RNA from strain SF370 served as control. The resulting strains, SF370Δ*speH* and SF370Δ*spy0129*, lacked the *speH* and *spy0129* genes, respectively.

### Biological assay: Pharyngeal cell adherence.

We tested late-logarithmic phase SF370Δ*speH* and SF370Δ*spy0129* mutants in an in vitro assay for adherence to Detroit 562 pharyngeal cells as previously described [15] to determine if either the *speH or spy0129* gene product was involved directly in the adherence of strain SF370 to pharyngeal cells. The parental strain SF370 served as control.

### Neighbor clustering.

We provide a general explanation of the principles of neighbor clustering followed by a more detailed explanation of the algorithms. Due to the large number of all putative clusters in the SF370 genome (~10^500^), we restricted our search space to clusters that are spatially related. During the assignment of neighbor clusters, we did not associate genes with functional annotations to prevent biasing the formation of clusters toward those that were “expected.” The GenomeCrawler algorithm, written in the statistical language R (http://www.R-project.org), steps through the expression data and identifies adjacent gene groupings that exhibit similar expression fold changes. The algorithm varies window size and applies a gap penalty for including in a cluster those genes that we did not observe experimentally to be present or genes that did not exhibit differential expression between sample and control.

GenomeCrawler calculates statistical significance of all putative resulting neighbor clusters (*P_K_* value), using a permutation algorithm with the sum of the *t*-test statistics (generated by Cyber-T) from each gene within a given cluster as the metric for comparison. We then inspected the output visually and disqualified groupings that violate the neighbor cluster definition based on established guidelines for functionally coupled gene pairs: genes occur on the same DNA strand and adjacent genes are separated by ≤300 bp [5]. We further restricted qualifying clusters to contain genes with a uniform direction of differential expression (i.e., all upregulated or all downregulated). Visual inspection is necessary because we have not yet had success at incorporating these specific parameters into the algorithms. To emphasize the importance of such inspections, we included the output prior to disqualifications for comparison (Table S4). We disqualified the following groupings: 491 contained genes located on different DNA strands; 127 contained adjacent genes separated by greater than 300 bp; and 24 contained genes that did not exhibit a uniform direction of expression. Since a specified gene could be a member of many different clusters, the cluster that generated the lowest *P_K_* value ≤ 0.05 and met all of the defined conditions of a neighbor cluster (as detailed in the text) is the one that we reported. The GenomeSpyer algorithm, also written in R, provides a method to view the GenomeCrawler output and to visualize clusters and their respective gene members. The GenomeSpyer plots of all datasets derived from this study can be found as Figures S2–S4.

### Theoretical basis for GenomeCrawler.

Conceptually, *P*_F_ reflects the physical change in gene expression between sample and control, whereas *P*_C_ reflects the significance of a gene in the context of a cluster and is based on combined information about genome structure (i.e., genome position) and activity (i.e., measured changes in expression). *P*_C_ reflects the cluster context and is not merely a recapitulation of the effect related by *P*_F_ for an individual gene, because on its own the *P*_F_ of a single gene is not sufficient to generate an informative *P*_C_ (i.e., *P*_C_ << 1). Validation of this point is found in the details of the algorithms implemented for calculating *P*_F_ and *P*_C_. The overall statistical significance of a specified gene, *g,* in regard to change in expression between sample and control is referred to as *P*_E_, and this probability is calculated as the product of two probabilities: *P*_F_, the *p-*value associated with observing the log_2_-fold change for the given gene, and *P*_C_, the *p-*value associated with the same given gene being a member of a specified cluster of genes. We treated this new probability as the posterior in Bayes' Theorem [22] and used the respective prior, likelihood, and cluster probabilities for its calculation. Calculation of the prior and likelihood used essentially the same algorithm for determining the cluster *P*_K_ value above, with the arguments of the prior and likelihood defining the respective set of *t*-test statistics to sum.

### GenomeCrawler algorithms and calculation of *p-*values.

*P*_E_ (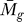 , *t_g_*, *K*), called the expression *P-*value and referred to as the *P*_E_ value, is equal to the product of two probabilities, *P*_F_ (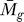 ) and *P*_C_ (*t_g_* | *K*), calculated with distinct permutation resampling algorithms (Equation 1):

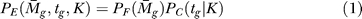



*P*_F_ (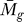 ) is the *p*-value associated with the log_2_-fold change in expression of a given gene (referred to as the *P*_F_ value). Its calculation uses the Westfall–Young stepdown permutation algorithm [19], where 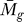 is the average log_2_-fold change of a specific gene and the basis set is the log_2_-fold change of a gene, M*_i,a_,* in which *i* is an element of the genes of the observable transcriptome and *a* is an element of the set of microarrays. The metric for comparison is *t_g_*, a Bayesian-derived regularized *t*-test statistic of the log_2_-fold change for the given gene [21]. *P*_C_ (*t_g_* | *K*) is the *p-*value that corresponds to the probability that a specific gene is a member of its assigned cluster. Calculation of *P*_C_ (*t_g_* | *K*) also uses *t*, but rather than a metric for comparison, it is the basis set for resampling composed of *t_i_* in which *i* is an element of the genes of the annotated genome. The metric for comparison is the sum of the elements of the set *K* = {t*_j_* : *j* ∈ *J*} in which *J* = {*j*: *j* ∈ {genes of the specified cluster}}. Since a gene can have membership in multiple clusters, our approach uses a dynamic windowing algorithm to sequentially search the genome for spatial clusters. The cluster that generates the lowest *P*_E_ (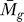 , *t_g_*, *K*) for the specified gene determines the reported value of *P*_E_ (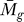 , *t_g_* , *K*).

Calculation of *P*_C_ (*t_g_* | *K*) relies on Bayes' Theorem (Equation 2) in which *P*_C_ (*t_g_*) is the prior, *P*_C_ (*K* | *t_g_*) is the likelihood, and *P*_C_ (*K*) is the probability associated with the cluster.

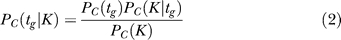



All of the right-hand side probabilities are readily calculated using the following general equation:

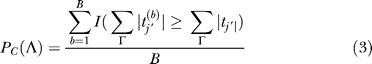
in which Λ ⊆ *K* and Λ = {t*_j_*_′_ : *j*′ ∈ Γ} in which Γ ⊆ *J* and represents a set of genes defined in the parenthesis of Equation 2. Since *P*_C_ (*K*) is the measure for the statistical significance of the specified cluster in our analysis, for this probability Γ = *J*. For the prior, Γ = {*j*′: *j*′ = *g*, ∃ ! *g* ∈ *J*}, whereas for the likelihood, Γ = {*j*′: *j*′ ∈ *J*, *j*′ ≠ *g*, ∃ ! *g* ∈ *J*). *B* is the total number of iterations of permutation resampling performed, with (*b*) representing a resampled value of the *b*th iteration. The indicator function *I*(·) equals 1 when the condition in parentheses is satisfied, and 0 when it is not.

### Relationship between *P*_F_ and *P*_C_.

We define the relationship between *P*_F_ (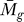 ) and *P*_C_ (*t_g_* | *K*), as both use *t_g_* for their respective calculations. For *t_g_* ≅ max t*_i_, P*_F_ (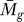 ) → 0 and 0 < *P*_C_ (*t_g_* | *K*) ≤ 1. Therefore, the analysis ensures that even the most significant gene with respect to *P*_F_ (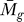 ) can theoretically have *P*_C_ (*t_g_* | *K*) = 1. For example, when a gene is a member of a cluster in which the other members are insignificant on a genome scale, *P*_C_ (*t_g_* | *K*) ≅ 1, since *P*_C_ (*K* | *t_g_*) ≅ 1 and *P*_C_ (*t_g_*) ≅ *P*_C_ (*K*). Conversely, for *t_g_* ≅ min t*_i_*, *P*_F_ (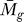 ) ≅ 1 and *P*_C_ ≅ 1, since *P*_C_ (*t_g_*) ≅ 1 and *P*_C_ (*K* | *t_g_*) ≅ *P* (*K*). Here, there is a strong dependency between *P*_F_ (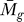 ) and *P*_C_ (*t_g_* | *K*). This prevents a gene with a relatively low *t_g_* value from being scored as significant due to a pure circumstantial association with a gene of *P*_F_ (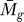 ) → 0. Hence, this analysis exhibits the required dynamic relationship between *P*_F_ (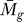 ) and *P*_C_ (*t_g_* | *K*) and, more important, is consistent with the criterion that, on its own, a gene with a low *P*_F_ (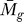 ) should not generate an informative *P*_C_ (*t_g_* | *K*) (i.e., *P*_C_ (*t_g_* | *K*) << 1). *P*_C_ (*t_g_* | *K*), therefore, reflects a group context derived from a cluster of genes and is not merely the recapitulation of the *P*_F_ (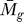 ) of an individual gene.

### Identification of putative operons.

The published SF370 genome does not contain promoter annotations, so we examined the entire genome in 100,000-bp segments (available for download at ftp://ftp.genome.ou.edu/pub/strep) and used the Vector NTI advance 9.0 sequence analysis suite (Invitrogen) to identify sequences that were similar (75% similarity threshold) to consensus streptococcal promoter sequences [64]. We cross-referenced the clusters containing a single upstream putative promoter sequence with a list of rho-independent terminator sequences, previously identified in the SF370 genome by TransTerm (www.tigr.org/software/transterm.html).

### Analysis of previously published streptococcal microarray data.

We analyzed recently published microarray data from S. pyogenes strain SF370 [57] in the same manner as the adherence data presented in this study to assess the overall reliability of our analytical methods. We applied the initial statistical package to assess the differential expression of individual genes, followed by the GenomeCrawler algorithms. We compared the results of this analysis, when applicable, to the published analysis of the array data.

### Software and microarray datasets.

For MIAME (Minimum Information About a Microarray Experiment) compliance, all microarray datasets (pre- and post-processing) have been deposited in the National Center for Biotechnology Information (NCBI) Gene Expression Omnibus (GEO; http://www.ncbi.nlm.nih.gov/geo) and given accession number GSE7620. Software to implement the GenomeCrawler and GenomeSpyer algorithms, as well as all corresponding datasets, are available for download at http://www.rockefeller.edu/vaf/streparray.php.

## Supporting Information

Figure S1 Comparison of Microarray and TaqMan Analyses (876 KB EPS)

Figure S2 Statistically Significant Type I Neighbor Clusters in the SF370 Genome (1.7 MB EPS)

Figure S3 Statistically Significant Type II Neighbor Clusters in the SF370 Genome (1.7 MB EPS)

Figure S4 Statistically Significant Type III Neighbor Clusters in the SF370 Genome (1.0 MB EPS)

Table S1 Summary of All Streptococcal Genes Exhibiting Differential Expression during Adherence to Pharyngeal Cells as Compared with Associated Streptococcal Control (138 KB XLS)

Table S2 DNA Primers Used in This Study (23 KB XLS)

Table S3 Fold Changes in Gene Expression Estimated Using Microarray and Real-Time Quantitative PCR (16 KB XLS)

Table S4 All Putative Neighbor Clusters Generated by GenomeCrawler (144 KB XLS)

Table S5 Putative Neighbor Clusters Following Visual Inspection (38 KB XLS)

Table S6 Summary of Neighbor Clusters Identified as Putative Operons through In Silico Inspection (21 KB XLS)

Table S7 Comparison of Log-Fold Changes of Genes Reported in a Previously Published Microarray Study of S. pyogenes SF370 (40 KB XLS)

Table S8 Summary of All Genes in a Mga-Deficient SF370 Mutant Exhibiting Differential Expression during Growth in Culture Broth Compared with the Parental SF370 Control (188 KB XLS)

Table S9 Putative Neighbor Clusters Identified by GenomeCrawler in Published Microarray Dataset from S. pyogenes SF370 (28 KB XLS)

### Accession Numbers

The GenBank (http://www.ncbi.nlm.nih.gov/Genbank) accession number for S. pyogenes strain SF730 (serotype M1) is AE004092.

The National Center for Biotechnology Information (NCBI) Gene Expression Omnibus (GEO; http://www.ncbi.nlm.nih.gov/geo) accession number for all microarray datasets in this paper is GSE 7620.

## Abbreviations

qRT: quantitative real time
RT: real time
